# Bioactive components of medicinal-food homology substances alleviating constipation via gut microbiota modulation: a review

**DOI:** 10.3389/fphar.2026.1880189

**Published:** 2026-08-11

**Authors:** Xinyuan Zhao, Yishan Song, Jiahui Zhu, Binghua Ma, Dong Liang, Zheng Xu, Yuhao Ma

**Affiliations:** 1 Centre for Translational Medicine Research, Naval Medical University, Shanghai, China; 2 College of Food Sciences and Technology, Shanghai Ocean University, Shanghai, China; 3 Department of Gastroenterology, The Third Affiliated Hospital of Naval Medical University (Shanghai Eastern Hepatobiliary Surgery Hospital), Shanghai, China

**Keywords:** emerging technology, functional constipation, gut microbiota, mechanism, medicinal-food homology

## Abstract

Functional constipation (FC) is a common gastrointestinal disorder with complex etiology. Clinically, it manifests as reduced bowel movement frequency, hard stools, straining during defecation, or incomplete evacuation, significantly impairing patients’ quality of life. While numerous constipation treatments are commercially available, they generally exhibit limited efficacy, pronounced adverse effects, and poor patient compliance. In recent years, the development and application of bioactive compounds from Medicinal-food homology (MFH) have garnered significant attention. Numerous studies indicate that various such compounds demonstrate marked efficacy in ameliorating FC. This paper systematically reviews the mechanisms by which bioactive substances from these plants alleviate FC through gut microbiota interactions, providing theoretical guidance for developing functional foods or health supplements with promising therapeutic effects for FC.

## 1 Introduction

Constipation represents a prevalent dysfunction within the digestive system. According to the Rome IV criteria, constipation is classified into four distinct subtypes: FC, irritable bowel syndrome with constipation (IBS-C), opioid-induced constipation (OIC), and functional defecation disorder (FDD) (Mearin et al., 2016). While these subtypes share overlapping symptoms like straining and hard stools, they possess distinct clinical boundaries (Corsetti et al., 2026). Specifically, IBS-C is differentiated from FC by the mandatory presence of recurrent abdominal pain linked to defecation (Sebastián Domingo, 2022). On the other hand, FDD is characterized by an anatomical or neuromuscular dysfunction of the pelvic floor, such as paradoxical anal contraction or inadequate propulsive force during defecation, leading to rectal outlet obstruction (Scott and Carrington, 2020). FC remains a diagnosis of exclusion when abdominal pain is absent and pelvic floor dyssynergia is not the primary driver (Bharucha and Lacy, 2020). Among these, FC has garnered considerable attention in recent years as a major subtype. Clinically, FC is characterized by decreased defecation frequency, the passage of dry and hard stools, difficulty during defecation, and a sensation of incomplete evacuation (Wang et al., 2024). Several researchers have suggested that the pathogenesis of FC is linked to delayed gastrointestinal motility and aberrant secretion of gastrointestinal hormones (Rao et al., 2016). The global prevalence of constipation ranges from 0.5% to 32.2%, affecting approximately 11%–20% of adults, with a notably higher incidence among females (Sperber et al., 2021), and up to 36% in elderly populations (Ge et al., 2017; Schiller, 2019). This pronounced female predisposition to FC can be attributed to a combination of hormonal, anatomical, and psychological factors (Chen et al., 2022). Physiologically, female sex hormones, particularly progesterone, have been shown to exert an inhibitory effect on colonic smooth muscle contraction by altering the enteric nervous signaling, thereby slowing down colonic transit (Alqudah et al., 2022; Lu et al., 2024). Anatomically, the female pelvic floor is highly susceptible to structural changes and neuromuscular micro-trauma during pregnancy and vaginal delivery, which may lead to pelvic floor dyssynergia or rectocele later in life (Mehrvarz et al., 2015). Additionally, a higher susceptibility to anxiety and emotional stress in females modulates the bidirectional gut-brain axis, further inducing visceral hypersensitivity and dysregulated gastrointestinal motility (De Palma et al., 2014). Furthermore, the age of onset has been progressively decreasing in recent years. Chronic constipation significantly impairs patients’ quality of life, and its complex etiology poses challenges for clinical management (Xu et al., 2022). Current therapeutic approaches for FC predominantly involve pharmacological interventions. However, these treatments exhibit limitations. Primarily, the multifactorial etiology of FC contrasts with the predominantly single-target mechanisms of existing drugs (Ferrada et al., 2020; Pizzino et al., 2017), thereby restricting overall efficacy, failing to achieve complete resolution, and often providing only transient symptom relief, which predisposes patients to recurrence (Johanson and Kralstein, 2007). Moreover, certain pharmacotherapies are associated with notable adverse effects, including drug dependence, intestinal injury, nausea, vomiting, and abdominal pain, particularly with prolonged use (Corsetti et al., 2021). Therefore, improving intestinal constipation and promoting intestinal and overall body health through non-pharmacological interventions and non-invasive treatment methods holds significant clinical translational value.

The human digestive tract hosts a complex microbial community, which is called the gut microbiota (Thursby and Juge, 2017). It consists of bacteria, fungi, viruses, archaea and protozoa. Among them, bacteria dominate, containing more than 2,000 species and 9.9 million genes that make up the vast majority of the gut microbiota (Tan et al., 2023). Bacteria in the gut microbiota can be categorized as pathogenic, beneficial and neutral based on their effect on the host. Pathogenic bacteria (about 10%) are capable of causing food-related intestinal diseases, food poisoning and other harmful conditions, such as *Clostridium perfringens*, *Prevotella spp*. and *Salmonella spp*. Beneficial bacteria (about 20%) such as *Clostridium butyricum*, *Ackermannia*, *Lactobacillus spp*. and *Bifidobacterium spp*, are beneficial to the host in the form of anti-inflammatory effects and inhibition of harmful bacteria. The remaining approximately 70% of bacteria are neutral, also known as opportunistic pathogens, and include *E. coli*, *Streptococcus*, and *Veronica* (Kim et al., 2019; Sebastián Domingo and Sánchez Sánchez, 2018)*.* To enhance presentation and provide a clear overview, the classification, representative taxa, and detailed physiological roles of these gut microbiota types are summarized in Table 1. The gut microbiota develops dynamically, changing from birth through childhood and becoming stable in adulthood. Its composition is influenced by factors such as genetics, geographic location, and antibiotic use, but diet is considered the most important factor. For example, feeding mice a high-fat diet for 24 h can modify their gut microbiota (Adak and Khan, 2019). Over thousands of years, the gut microbiota and humans have co-evolved, establishing a mutually beneficial symbiotic relationship essential for gut health (Thursby and Juge, 2017). The microbiota performs various functions, including maintaining the intestinal mucosal barrier, breaking down complex nutrients like dietary fiber, producing metabolites such as Short-chain fatty acids (SCFAs), preventing the growth of harmful bacteria, regulating both intestinal and systemic immune balance, influencing hormonal responses, and eliminating foreign drugs and toxins (Dong et al., 2019; Li et al., 2020). An imbalance in microbial composition and reduced diversity, known as dysbiosis, not only causes damage to intestinal health but also inflicts harm on the body’s metabolism and the development of the nervous system.

**TABLE 1:** Classification, representative taxa, and physiological impacts of different types of gut microbiota.

Microbiota category	Estimated proportion	Representative taxa	Primary physiological impacts & roles in gut health
Beneficial bacteria (Shen et al., 2022)	20%	*Clostridium butyricum, Akkermansia, Lactobacillus spp, Bifidobacterium spp.*	Anti-inflammatory propertiesPathogen inhibitionSCFAs production & mucosal homeostasis
Detrimental Bacteria (Fekete and Buret, 2023)	10%	*perfringens, Prevotella spp., Salmonella spp.*	Foodborne pathogen infection Intestinal toxicity & inflammation Microbiological dysbiosis
Neutral Bacteria (Zeng et al., 2017)	70%	*Escherichia coli, Streptococcus, Veronica*	Symbiotic under normal homeostasisPathogenic transformation upon dysbiosis

In recent years, there has been a growing global consumer preference for health solutions that are natural, safe, and sustainable (Wang et al., 2025). Within this context, the concept of MFH has established significant institutional advantages in China, subsequently extending its influence internationally. This development has engendered a dual-driven growth model characterized by robust domestic demand alongside increasing international interest. Since the initial publication of the Catalogue of Substances That Are Both Food and Medicine in 2002, China’s National Health Commission has expanded this list by adding nine substances in 2023, thereby increasing the total to 106 items and providing the industry with a legally compliant resource base (Chen et al., 2023). On the international stage, MFH remains primarily at the conceptual introduction phase; however, collaborative efforts focusing on high-quality raw materials and advanced technologies have begun to facilitate breakthroughs. Notably, the year of 2024 International Functional Food Conference featured a dedicated session on MFH composite polysaccharides (Gao et al., 2018), marking the integration of MFH into the global discourse on functional food technologies through the lens of polysaccharide-mediated immunomodulation (Jach et al., 2022; Micucci et al., 2024). Within this evolving framework, research efforts are increasingly concentrated on innovation and development in the field of medicinal foods, aiming to create high-value functional foods and health products by synthesizing traditional medical knowledge with contemporary technological advancements (Huang et al., 2025). Scholars are expanding their focus beyond the extraction and utilization of individual components to encompass the study of compound formulations (Gao et al., 2018; Micucci et al., 2024), with the objective of achieving more holistic and comprehensive health benefits.

MFH substances possess dual nutritional and medicinal properties, being safe for consumption as part of the daily diet by healthy individuals whilst also exerting therapeutic effects during illness. Their safety and efficacy have been thoroughly validated through millennia of medical practice and ancient medical literature in China (Tan et al., 2023; Yang et al., 2016). Modern research further reveals that MFH foods are rich in diverse bioactive components. Through complex molecular mechanisms involving multiple targets and pathways, they can systematically intervene in pathological processes such as oxidative stress, chronic inflammation, and metabolic disorders (Gong et al., 2020). Among these, “modulation of the gut microbiota” is widely recognized as one of the core mechanisms (Tan et al., 2023). MFH primary components include polysaccharides, phenolic compounds, and dietary fiber. Notably, within the MFH framework, no absolute boundary exists between drugs and food (Zuo et al., 2023), a characteristic providing theoretical grounding for its precision nutritional intervention in gastrointestinal disorders. Recent empirical studies demonstrate that polysaccharides extracted from *Astragalus* membranaceus root markedly increase fecal water content and intestinal transit time in murine models of FC (Liu X. et al., 2022). Additionally, phenolic compounds such as Mogroside V (Wu et al., 2022) and total mulberry flavonoids (Lee et al., 2017) suppress the proliferation of potentially pathogenic bacteria while promoting the enrichment of *Lactobacillus* species. Dietary fibers like *hawthorn* pectin (Zhang et al., 2024) and resistant starch from coix seed (Wu G. et al., 2025) further contribute to the restoration of intestinal barrier integrity and accelerate gastrointestinal transit by elevating SCFAs concentrations and enhancing mucus secretion from goblet cells. These MFH-derived functional factors exert their prokinetic effects through synergistic interactions along the microbe-metabolite-enteric nervous system axis, thereby providing novel molecular insights and viable candidate formulations for dietary interventions targeting constipation. In this review, we have summarized the association and underlying mechanisms between constipation and microorganisms. We have classified the types of MFH and analyzed the specific mechanisms by which MFH regulates intestinal peristalsis through the gut microbiota. Additionally, we have summarized the latest advancements in microbial technologies for constipation treatment based on MFH, aiming to provide a theoretical foundation for the development of functional foods, nutritional supplements, and novel therapeutic approaches.

## 2 Constipation and the gut microbiota

### 2.1 Relationship between FC and gut microbiota

The gut microbiota refers to the diverse microbial communities residing within the human digestive system, increasingly recognized as a key factor in facilitating interactions between different bodily systems (Luan et al., 2018). The formation of gut microbial communities, comprising diverse bacteria, fungi, viruses, and other microorganisms, is vital for maintaining intestinal homeostasis, host health, and disease resistance (Barbara et al., 2005). Disruption of these communities, commonly termed dysbiosis, is increasingly associated with numerous disorders, including functional gastrointestinal diseases such as irritable bowel syndrome (Zhao and Yu, 2016) and constipation (Canani et al., 2011). To date, several studies have examined the gut microbiota of children with constipation (Spencer and Hu, 2020). Scientists employed Quantitative polymerase chain reaction (qPCR) to analyse seven lactic acid bacteria in faecal samples from 40 children with FC and 40 healthy controls (Erhardt et al., 2023). Results indicated significantly lower total *Lactobacilli* counts in the FC group compared to controls (Kaur et al., 2019). Similarly, another study employing polymerase chain reaction (PCR) to quantify faecal microbiota again demonstrated markedly reduced abundance of the *Lactobacillus* genus in constipated children relative to non-constipated controls. Among constipated children, increased abundance of *Ascomycota phylum* species was observed, alongside elevated levels of *Anaplasma*, *Paraplasma*, and *Bifidobacterium longum*, while levels of *Alipis* and *Rumen subphylum* were reduced (Steed et al., 2010). With continuous technological advancements, high-throughput sequencing methods such as 16S rRNA sequencing have greatly facilitated gut microbiome analysis. In a recent study, Zhu’s team pioneered the systematic analysis of the 16S rRNA gene using pyrophosphate sequencing (Zhou et al., 2024). Their findings revealed that obese constipated children, typically exhibiting insufficient dietary fiber intake, displayed a significantly reduced relative abundance of *Prevotella* in their gut microbiota. Concurrently, bacteria capable of producing butyrate, such as *Faecalibacterium*, *Roseburia*, and *Faecalibacterium* species, were more abundant in the affected group than in healthy controls (Zhao and Yu, 2016). Separately, Kaur et al. (2019) analysed the gut microbiota of adults. Researchers employed 16S rRNA sequencing and metabarcoding to analyse the gut microbiota of adults with and without FC. Findings indicated that bacterial genera *Bacteroides*, *Roseburia*, and *Coprococcus* were negatively correlated with constipation, whereas *Faecalibacterium* showed a positive association with the condition.

These studies indicate that the microbiota plays a significant role in the regulation of intestinal constipation. Beneficial bacteria such as *Lactobacillus* may alleviate constipation by promoting intestinal peristalsis. This underscores the potential of adopting a microbiota-focused perspective as a valid approach for investigating disease characterization.

### 2.2 Characterization of the gut microbiota of the FC population

Previous research has indicated that patients with constipation exhibit a significant increase in the abundance of *Actinobacteria* and a notable decrease in *Proteobacteria* compared to healthy individuals (Li et al., 2023; Zare et al., 2018). Luan et al. (2018) conducted a study using a mouse model to investigate chronic constipation-induced intestinal cancer. Their findings revealed that FC substantially raises the risk of developing intestinal tumors and causes significant alterations in the gut microbiota. In normal flora, *Lactobacillus* was the predominant bacterial group, but its abundance dropped markedly from 70% to 45% in the constipation model group, and further declined to less than 10% in the intestinal cancer model group. Conversely, *Barnesiella* accounted for 5% of the normal flora, increased to 8% in the constipation model group, and surged to 40% in the bowel cancer model group. This study illustrates the dynamic fluctuations in the composition of the gut microbiota throughout the progression from healthy individuals to those experiencing constipation, and further to complications such as constipation-associated colorectal cancer. These changes are primarily characterized by an increased prevalence of pathogenic bacterial species.

The gut microbiota influences intestinal motility through a variety of mechanisms. Barbara et al. (2005) demonstrated that end-products produced by bacterial fermentation are able to regulate intestinal motility through mediators released by intestinal neuroendocrine factors and the intestinal immune response. Among them, SCFAs, as the main products of bacterial anaerobic metabolism, play an important role in the regulation of intestinal motility. Studies have demonstrated that SCFAs stimulate propulsive contractions of the ileum and appear to directly stimulate contractions of ileal and colonic smooth muscle (Zhao and Yu, 2016). From a quantitative pharmacology perspective, the translation of these microecological mechanisms into clinical efficacy depends heavily on specific dose-response thresholds within the intestinal lumen. Human physiological and clinical mapping indicates that the total concentration of SCFAs in a healthy colonic lumen typically ranges from 70 to 130 mM (with acetate, propionate, and butyrate in an approximate molar ratio of 60:20:20) (Suzuki et al., 2008). To directly trigger propulsive smooth muscle contractions and effectively activate free fatty acid receptors 2 and 3 (FFAR2/3), local luminal butyrate concentrations must consistently reach a therapeutic threshold of 10–20 mM (Dalile et al., 2019). Below these millimolar ranges, the activation of the enteric nervous system (ENS) and the subsequent upregulation of aquaporins remain insufficient to alter fecal water content or accelerate colonic transit times (Dalile et al., 2019; Pham et al., 2018). To achieve these therapeutically effective luminal butyrate and total SCFAs levels, translational dietary interventions require strict dose control. Clinical guidelines and dose-response trials demonstrate that a daily intake of 25–35 g of total dietary fiber, or targeted supplementation with 10–15 g/day of highly fermentable soluble prebiotics (such as inulin, fructooligosaccharides, or specific MFH-derived polysaccharides), is required to significantly elevate colonic butyrate production above the constipation baseline (Micka et al., 2017). Specifically, every 10 g increase in soluble dietary fiber intake correlates with an incremental rise in luminal SCFAs concentration by approximately 15%–20%, thereby facilitating the mechanical translation from microbial fermentation to clinical laxative outcomes (Medawar et al., 2021). In addition, SCFAs, especially butyrate, exhibit Chloride ion (Cl^−^) absorption-promoting and antisecretory effects, which contribute to the regulation of water and electrolyte homeostasis in the intestinal tract, which in turn affects fecal water content and hardness (Canani et al., 2011). The ENS represents the largest component of the peripheral nervous system and primarily functions to regulate gastrointestinal digestion. The ENS modulates intestinal activity through the release of various neurotransmitters from its neuronal network, which are broadly categorized into excitatory and inhibitory types. Excitatory neurotransmitters, such as acetylcholine (ACh) and 5-hydroxytryptamine (5-HT), facilitate the contraction of intestinal smooth muscle and promote peristaltic movements. Conversely, inhibitory neurotransmitters, including vasoactive intestinal peptide (VIP) and nitric oxide (NO), suppress gastrointestinal motility and reduce peristalsis. Thus, the ENS orchestrates gastrointestinal function by balancing excitatory and inhibitory neurotransmitter signaling, thereby regulating nerve conduction and enhancing gastrointestinal motility, which can contribute to the alleviation of constipation symptoms (Spencer and Hu, 2020).

Given the pivotal role of gut microbiota dysbiosis in the pathophysiology of constipation, improving the symptoms of constipation by regulating the flora has become an important therapeutic direction (Zhou et al., 2024). In this context, the concept of MFH, which is rooted in traditional Chinese medicine (TCM), provides a unique perspective and strategy for the prevention and treatment of constipation. Medicinal substances are usually rich in dietary fiber, oligosaccharides and other bioactive components, which can act as prebiotics to selectively promote the growth and metabolic activities of beneficial gut microbiota (e.g., *Bifidobacterium spp*. and *Lactobacillus spp*.) (Erhardt et al., 2023). By optimizing the flora structure and restoring the intestinal microecological balance, the production of SCFAs is then increased, thus effectively improving the intestinal motility function. Adequate SCFAs not only stimulate intestinal smooth muscle contraction and promote intestinal peristalsis, but also help to regulate intestinal water and salt absorption and improve fecal traits, thereby relieving constipation (Kaur et al., 2019). In addition, some of the medicinal food ingredients may also possess anti-inflammatory, antioxidant and intestinal barrier repair functions, which help to reduce intestinal inflammation and barrier damage, further improve the intestinal environment, and create favorable conditions for normal intestinal motility (Steed et al., 2010). Therefore, applying the concept of medicinal food to constipation management is expected to fundamentally improve gut microbiota dysbiosis through a multi-targeted and synergistic approach, thereby effectively alleviating constipation symptoms and enhancing the overall health of patients.

## 3 The mechanism by MFH regulates gut microbiota to improve constipation

**FIGURE 1:**
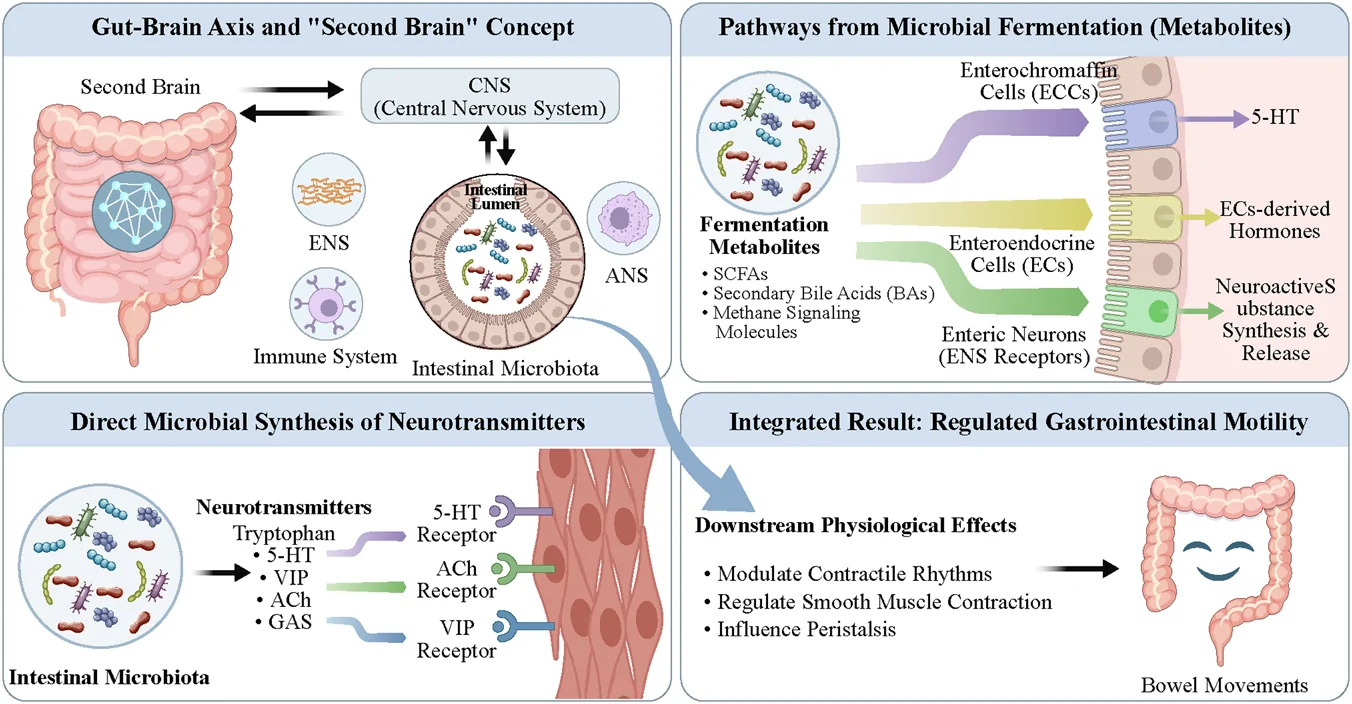
Mechanisms of gastrointestinal function regulation by the gut microbiota via neuroendocrine-immune pathways. Gut-Brain Axis and the “Second Brain” Concept: Illustrates the multidimensional, bidirectional communication networks connecting the gut microbiota with the CNS, ENS, ANS, and the mucosal immune system within the intestinal lumen. Pathways from Microbial Fermentation (Metabolites): Depicts how bacterial fermentation products (such as SCFAs and BAs) activate specific receptors on ECCs and ECs, triggering the synthesis and secretion of 5-HT and other neuroactive substances. Direct Microbial Synthesis of Neurotransmitters: Shows the capacity of the intestinal microbiota to autonomously synthesize key neurotransmitters (including tryptophan, 5-HT, VIP, ACh, and GAS) that bind directly to their corresponding receptors (5-HT, ACh, and VIP receptors) on smooth muscle cells or enteric neurons. Integrated Result of Regulated Gastrointestinal Motility: Represents the downstream physiological effects, including the modulation of contractile rhythms, regulation of smooth muscle contraction, and steering of intestinal peristalsis to promote regular bowel movements. The rhythmic contractions and peristalsis of the intestines are jointly regulated by these signals.

An autonomous neural-immune-endocrine regulatory network is present within the intestinal lumen, leading to the characterization of the intestine as the “second brain.” This bidirectional communication system, termed the “gut-brain axis,” encompasses the Central nervous system (CNS), ENS, Autonomic nervous system (ANS), as well as the intestinal microbiota and its metabolites (Zhao et al., 2021). Notably, the microbial community serves as a pivotal component in maintaining homeostasis across gastrointestinal, central, and peripheral neural and immune systems. Functionally, metabolites produced by microbial fermentation, such as SCFAs, secondary bile acids (BAs), and methane signaling molecules—can activate enterochromaffin cells (ECCs), enteroendocrine cells (ECs), and specific receptors on intestinal neurons. This activation promotes the synthesis and release of neuroactive substances, thereby precisely regulating intestinal motility (Wang and Yao, 2021). Furthermore, the intestinal microbiota synthesizes various neurotransmitters, including tryptophan, 5-HT, VIP, ACh, and gastrin (GAS). These neurotransmitters modulate the contractile rhythms of smooth muscle by interacting with specific receptors, ultimately influencing intestinal peristalsis and bowel movements (Heiss and Olofsson, 2018). Collectively, these findings underscore the central role of the intestinal microbiota in the integrated neuro-endocrine-immune regulation of gastrointestinal function. The following Figure 1 illustrates the relevant mechanism of action.

### 3.1 Gut microbiota through metabolites treating constipation

SCFAs, predominantly acetic acid, propionic acid, and butyric acid, represent key end-products of the anaerobic fermentation of undigested dietary fibers by intestinal microbiota. These metabolites are generated throughout the gastrointestinal tract, spanning from the proximal small intestine (jejunum and ileum) to the distal colon (descending colon, sigmoid colon, and rectum) (van der Hee and Wells, 2021). As central components of the bacterial metabolic network, SCFAs contribute to microecological regulation by acidifying the intestinal lumen, thereby inhibiting the colonization and proliferation of pathogenic or opportunistic bacteria-such as *Escherichia coli, Staphylococcus*, and *Salmonella*-that exhibit low acid tolerance (Nicholson et al., 2012). Experimental animal models have further demonstrated that administration of *Bifidobacteria* in loperamide-induced constipated mice results in elevated colonic SCFAs concentrations, reduced luminal pH, enhanced smooth muscle contractility and propulsion rates, and significant amelioration of defecation impairments (Wang et al., 2017a). Epidemiological studies corroborate these findings, indicating that diets enriched with fermentable dietary fibers increase the α-diversity of gut microbiota and augment SCFAs production relative to Western diets characterized by high fat and sugar content (García-Vega Á et al., 2020). *In vitro* fecal fermentation assays conducted by Tuncil et al. using arabinoxylan derived from various wheat bran sources revealed that variations in the microstructural properties of dietary fibers significantly influence both bacterial community composition and metabolic output profiles. Clinically, analyses of fecal samples from 30 patients with persistent chronic constipation demonstrated reduced total SCFAs and butyric acid levels compared to healthy controls (Shi et al., 2016). Subsequent dietary supplementation with soluble fiber in these subjects was associated with increased colonic butyric acid concentrations, shortened gastrointestinal transit times, and thickening of the mucus layer (Zhuang et al., 2019). These effects are hypothesized to arise from fiber-induced stimulation of peptide hormone secretion, including glucagon-like peptide-1 (GLP-1) from colonic L-cells, alongside upregulation of tight junction protein expression, thereby enhancing intestinal barrier integrity (Müller et al., 2020). Currently, strategies aimed at optimizing dietary fiber type and intake to promote SCFAs production are considered promising microecological interventions for the alleviation of functional constipation.

BAs, the principal bioactive constituents of bile, are synthesized by hepatocytes from cholesterol through both classical and alternative metabolic pathways (Li et al., 2023). These BAs are secreted into the intestinal lumen, where they facilitate lipid digestion and absorption, modulate intestinal motility, and inhibit microbial overgrowth via multiple mechanisms, thereby preserving the homeostasis of the intestinal mucosal barrier. Specifically, BAs activate G protein-coupled bile acid receptor 1 (TGR5) and transient receptor potential A1 (TRPA1) receptors located on the surface of intestinal chromaffin cells, stimulating the release of 5-HT. This release subsequently enhances smooth muscle contraction and colonic fluid secretion, contributing to the maintenance of normal gastrointestinal transit (Bajor et al., 2010). Moreover, BAs serve a critical “gatekeeper” function in maintaining microecological balance by selectively promoting the colonization of probiotic bacteria while suppressing the proliferation of pathogenic species. Notably, BAs deficiency is frequently observed in pathological states characterized by dysbiosis and impaired intestinal motility (Quigley and Spiller, 2016). In a murine model of loperamide-induced constipation, oral administration of *Bacillus subtilis* markedly ameliorated defecation dysfunction. Metabolomic analyses demonstrated an increased colonic BAs pool alongside elevated 5-HT levels released from enterochromaffin cells via the TGR5/TRPA1 signaling pathway, implicating the BAs-microbiota-5-HT axis in mediating prokinetic effects (Zhan et al., 2021). Additional research has revealed that apple juice may also alleviate constipation by downregulating the expression of BAs transporter proteins, such as the apical sodium-dependent bile acid transporter (ASBT), in the ileum and colonic apical membrane. This downregulation reduces BAs reabsorption through the enterohepatic circulation, thereby sustaining higher luminal BAs concentrations and enhancing intestinal peristalsis (Zhu et al., 2023). Therefore, these findings suggest that modulation of BAs synthesis, transport, and receptor-mediated signaling represents a promising integrated microecological and metabolic therapeutic strategy for the management of FC.

In addition to the short-chain fatty acids and bile acids mentioned above, methane (CH_4_) and hydrogen sulfide (H_2_S), gaseous metabolites released during fermentation by gut microbiota, also have bi-directional modulatory potential for gastrointestinal motility (Triantafyllou et al., 2014). CH_4_ may retard content transport by interfering with neurotransmitter signaling, Accumulating evidence underscores that H_2_S does not merely act as a unidirectional inhibitor; rather, it exerts a sophisticated bidirectional regulatory role on intestinal motility governed by a distinct concentration-dependent threshold (Verbeure et al., 2021). At low physiological concentrations (typically in the nanomolar to low micromolar range), functions as a prokinetic agent that actively promotes intestinal smooth muscle contraction (Quan et al., 2015). Mechanistically, low-dose achieves this by inhibiting voltage-gated potassium channels H_2_S to induce membrane depolarization, or by modulating intracellular mobilization via -type calcium channels and stimulating endogenous cholinergic neurotransmission (Jimenez, 2010; Quan et al., 2015). Conversely, when reaches high concentrations within the colonic lumen-often driven by an overgrowth of sulfate-reducing bacteria-it switches to an inhibitory role, inducing smooth muscle relaxation and reducing contraction amplitude primarily through the activation of ATP-sensitive potassium channels (Brady and Reay, 2025). Therefore, MFH interventions targeting the gut microbiota must precisely balance -producing bacterial taxa to maintain this gasotransmitter within its prokinetic physiological window. 5-HT synthesized by intestinal chromaffin cells is the key excitatory transmitter that initiates the peristaltic reflex, in contrast to local dopamine (DA) release that exhibits an inhibitory effect. A clinical study by Villanueva-Millan et al. (2022) showed that there was no significant difference in serum 5-HT levels between the methane expiratory-positive group and the negative group of patients with IBS in the fasted state. However, the after a standardized test meal, 5-HT peaks were significantly lower in the breath-positive than in the negative group, suggesting that methane may impair the postprandial 5-HT response. Recent animal experiments have also confirmed that elevated intestinal luminal methane concentrations and synchronized decreases in colonic propulsion rates in constipation model mice further support methane-mediated kinetic inhibition (Vega et al., 2015). On the other hand, H_2_S, generated by the conversion of exogenous sodium hydrosulfide via the sulfide reduction pathway of gut microbiota, can cause membrane hyperpolarization through activation of smooth muscle ATP-sensitive potassium channels (KATP), which is manifested as dilatation of the intestinal tubes with reduced peristalsis (Deplancke and Gaskins, 2003). Therefore, gaseous signaling molecules in the metabolites of the flora may be involved in the development and progression of functional constipation either by modulating the 5-HT/DA balance or by acting directly on smooth muscle.

### 3.2 Gut microbiota regulate the nervous system

In recent years, the novel concept of the brain-gut-bacteria axis (BGBA) has been introduced, providing valuable insights into the pathogenesis of FC. The brain-gut axis (BGA) constitutes a bidirectional communication pathway linking the gastrointestinal tract and the nervous system, encompassing the CNS, ANS, ENS, and the hypothalamic-pituitary-adrenal (HPA) axis. This axis interacts with the gut microbiota to modulate intestinal motility (Zhao et al., 2021). Within the nervous system-mediated regulation of intestinal motility by the gut microbiota, the ENS plays the most critical role, followed by the ANS and then the CNS (Dimidi et al., 2017). The excitatory neurotransmitter 5-HT in the ENS is implicated in the modulation of intestinal motility by influencing the rate of peristalsis. Empirical evidence indicates that intestinal chromaffin cells, in the presence of *Clostridium perfringens*, stimulate 5-HT secretion, thereby enhancing intestinal motility (Yano et al., 2015). Furthermore, research has demonstrated that *Bifidobacterium bifidum* ameliorates constipation by upregulating the expression of 5-HT receptor 4 (5-HT_4_R) in the intestinal mucosa of constipation model mice, consequently promoting intestinal peristalsis. Additionally, NO an inhibitory neurotransmitter within the ENS, has been shown to activate soluble guanylate cyclase upon elevation in serum levels, resulting in decreased intracellular Ca^2+^ concentrations (Kunze et al., 2009). This cascade induces the relaxation of intestinal smooth muscle and impairs gastrointestinal peristalsis. Notably, within the STC subphenotype of FC, this inhibitory neurosignaling is often hyperactivated, contributing to the severe deceleration of colonic transit. Moreover, animal studies have revealed that probiotic or prebiotic administration can rectify dysbiosis in the host colon, restore afferent nerve function responsible for colonic motility control, and thereby ameliorate the delayed transit characteristic of STC within the overarching pathological framework of FC. Accumulating multi-omics data indicate that the gut microbiota, via its characteristic metabolites such as SCFAs and BAs, can stimulate enterochromaffin cells to release 5-HT, thereby promoting colonic propulsive motility (D’Ascola et al., 2018). The activation of the secretory-peristaltic reflex cascade necessitates the binding of 5-HT to the 5-HT_4_R, selective 5-HT_4_R agonists, including procaine, have demonstrated efficacy in alleviating functional constipation (Daniali et al., 2019). Enhanced activity of tryptophan hydroxylase 1 (TPH1), the rate-limiting enzyme responsible for enteric 5-HT synthesis, further potentiates these effects. Experimental studies in animal models revealed that oral administration of butyric acid derivatives markedly accelerated colonic transit in TPH1 wild-type mice, whereas this effect was abolished in TPH1 knockout mice, thereby substantiating the pivotal mediating role of 5-HT in regulating colonic motility (Vincent et al., 2018). Beyond the 5-HT pathway, multiple gastrointestinal hormones and neurotransmitters contribute to the complex network governing colonic motility. Motilin (MTL) exerts dual functions by stimulating gastric acid secretion and enhancing peristalsis; clinical investigations have reported significantly reduced serum MTL levels in children with constipation compared to healthy controls, implicating MTL deficiency as a potential mechanism underlying slow-transit constipation in pediatric populations (Ulusoy et al., 2021). Additionally, Substance P (SP) and VIP are frequently downregulated in colonic tissues of constipated patients. SP plays a critical role in modulating colonic dynamics by inhibiting inflammatory factor release and reinforcing epithelial tight junctions, thereby enhancing mucosal barrier integrity. Rather than functioning as a unidirectional prokinetic or inhibitory factor, VIP plays a multi-target, bidirectional regulatory role in the pathogenesis of functional constipation through three distinct physiological arms (Bai et al., 2024). First, within the myenteric plexus, VIP is released by inhibitory motor neurons to directly inhibit smooth muscle contraction, which reduces basal tone and limits excessive colonic spasms-a mechanism that can decelerate gastrointestinal transit when overexpressed (Sarna, 2010). Second, VIP simultaneously acts on receptors in the submucosal plexus and mucosal epithelial cells to promote robust fluid and electrolyte secretion into the colonic lumen, which acts as a natural stool softener to ease evacuation (Burleigh and Banks, 2007). Third, VIP exerts powerful anti-inflammatory effects by suppressing pro-inflammatory cytokines and protecting the ENS from oxidative damage (Delgado and Ganea, 2013). Therefore, the net clinical effect of VIP on constipation represents a homeostatic balance: while its smooth-muscle-relaxing property may slow down transit, its pro-secretory and anti-inflammatory attributes actively counter the dry, hard stool and mucosal barrier defects characteristic of FC (Wang Y. et al., 2023). MFH ingredients that modulate the gut microbiota can fine-tune endogenous VIP levels to exploit this bidirectional equilibrium. Conversely, somatostatin (SS), an inhibitory neuropeptide, induces smooth muscle relaxation and prolongs intestinal transit time (Tian et al., 2023), whereas the excitatory neurotransmitter ACh synergistically augments contractile and secretory functions. Notably, a recent study demonstrated that administration of a novel *Lactobacillus* strain significantly increased defecation frequency and fecal water content in constipated mice; this effect was closely associated with upregulation of serum MTL, SP, ACh, and VIP levels alongside concurrent downregulation of SS concentration (Tan et al., 2021). Collectively, these findings elucidate the integrated role of the gut microbiota-metabolite-neurotransmitter axis in the pathogenesis and therapeutic modulation of constipation, providing a theoretical foundation for microecology-based neurohormonal combination treatment strategies.

**FIGURE 2:**
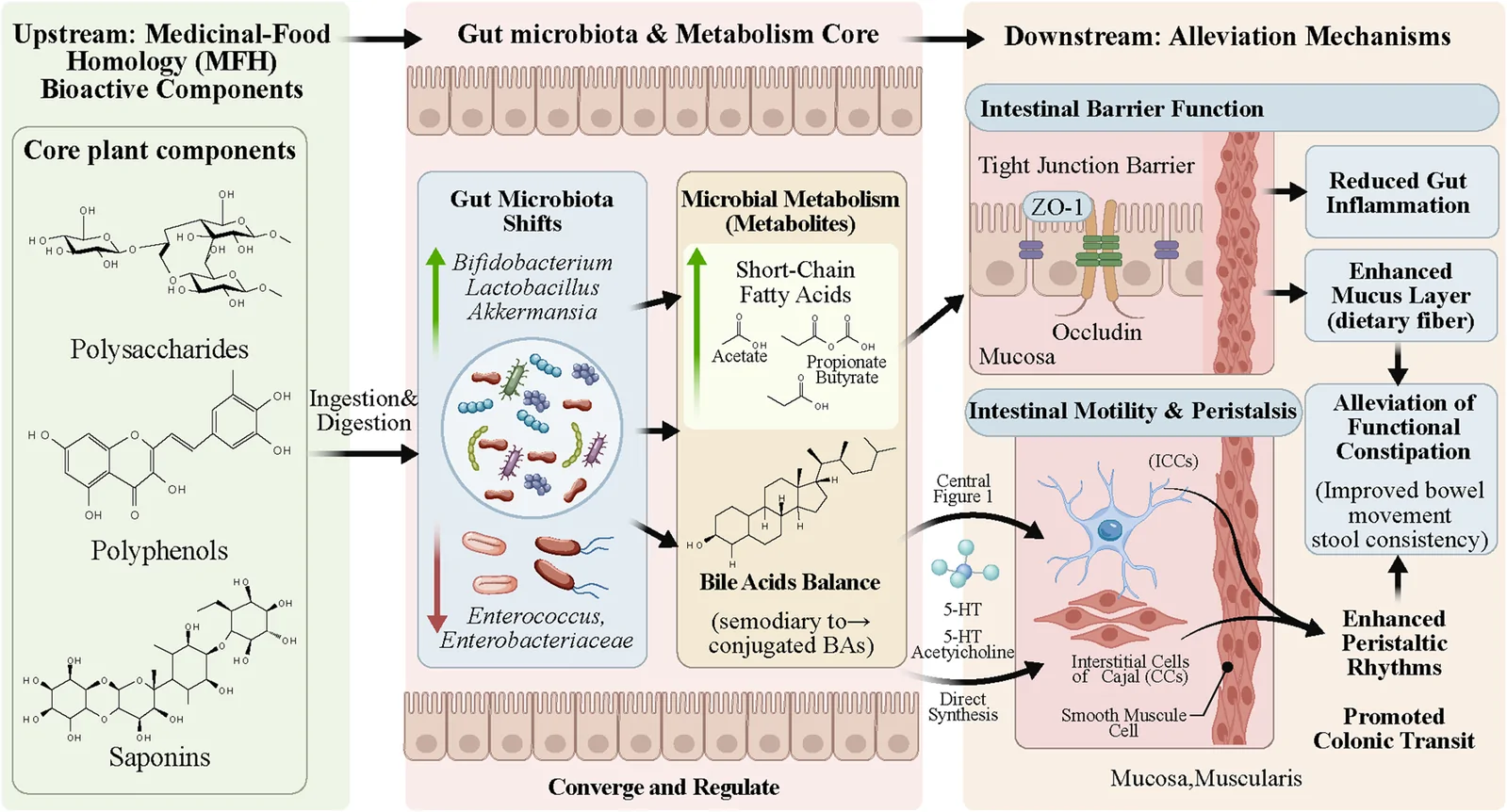
Schematic diagram illustrating the mechanisms of bioactive compounds from MFH in alleviating FC via gut microbiota modulation. The active compounds reshape the microbial community, stimulate the production of beneficial metabolites (such as SCFAs), protect the intestinal epithelial barrier, and ultimately enhance colonic peristalsis through the neuro-smooth muscle network.

To further elements the clarity and systematically understand the multi-dimensional therapeutic effects of MFH, the comprehensive interactive networks between specific bioactive compounds and gut microbiota are systematically summarized in Figure 2. Briefly, these functional factors exert prokinetic benefits via three major dimensions: microbial community modulation, metabolic pathway regulation, and the restoration of intestinal barrier & motility.

## 4 Classification of MFH and research advances in improving gut health

The concept of MFH, deeply rooted in TCM, refers to a unique category of natural resources that possess dual attributes as both daily nutrients and therapeutic agents (Ng et al., 2023). To address the historical ambiguity of these substances, the National Health Commission (NHC) of China has legally codified a compliant resource base (Ng et al., 2023; Zhang et al., 2022). However, from the perspective of modern food science and chemical taxonomy, the classification of MFH substances with anti-constipation efficacy warrants a more precise and rigorous framework rather than an overly broad generalized definition (Liu Y. et al., 2022). Based on the chemical structures and functional properties of the primary active ingredients documented to date, the anti-constipation MFH factors can be systematically categorized into three major domains: Carbohydrates (encompassing macromolecular polysaccharides and dietary fibers) (Gong et al., 2024), Phenolic Compounds (Rudrapal et al., 2025), and Quinones (specifically anthraquinones) (Liu Y. et al., 2022). This review restricts its scope primarily to those NHC-approved MFH materials whose specific molecular fractions have been validated through preclinical or clinical microecological evidence (Wu J et al., 2025). Some evidence indicates that representative MFH compounds—including *Dendrobium officinale*, *Tagetes erecta*, and *Foeniculum vulgare*—improve gastric function via the gut-myenteric microbiota axis: *Dendrobium* remodels microbial communities while stimulating gastric acid secretion, enhancing peristalsis, and inhibiting gastric mucosal injury (Birdane et al., 2007; Ma et al., 2022); *Luteolin* from *marigold* accelerates healing of acetic acid-induced gastric ulcers and reduces recurrence rates through antioxidant and anti-inflammatory activities (Meurer et al., 2022); trans-anethol and carvone in *fennel* leaves alleviate functional dyspepsia, flatulence, and diarrhoea via gastric protective mechanisms (Meurer et al., 2022). Furthermore, lignin-rich plant fibers such as *betel nut seeds*, *milk thistle fruit*, and flaxseed act as prebiotics, selectively promoting beneficial bacterial proliferation to further stabilise the microbiological barrier and alleviate gastrointestinal symptoms including constipation (Thumann et al., 2019). Therefore, we will examine the effectiveness of MFH as a therapeutic approach for FC. Research has shown that medicinal substances typically owe their effectiveness to certain active compounds, which are generally categorized as carbohydrates, phenols, and (Cardona et al., 2013; Khan et al., 2006; Lear et al., 2019; Wu et al., 2016) quinones and anthraquinones.

### 4.1 Carbohydrates

Carbohydrates constitute the most fundamental biomolecules regulating the intestinal microenvironment. It is worth noting that within classical literature, “polysaccharides” and “dietary fiber” are frequently discussed in parallel; however, from a structural standpoint, dietary fiber is essentially a functional subclass of non-digestible carbohydrates, primarily consisting of non-starch polysaccharides and oligosaccharides (Lovegrove et al., 2017). To clarify their hierarchy, we divide this section into macromolecular polysaccharides and specific fiber fractions.

Natural non-digestible polysaccharides are extensively distributed across MFH plants and microbes. These macromolecules bypass upper gastrointestinal digestion and serve as selective substrates that increase the -diversity of the gut microbiota, boosting the relative abundance of beneficial genera like *Lactobacillus* and *Bifidobacterium* (Ge et al., 2017). Mechanistically, their fermentation elevates SCFAs production, which acidifies the colonic environment, downregulates aquaporin 4 (AQP4) and aquaporin 8 (AQP8) expression, and uregulates aquaporin 3 (AQP3) to precisely balance fecal water metabolism (Gao et al., 2024). For instance, polysaccharides extracted from *Astragalus* membranaceus root and *Platycodon* grandiflorum have shown profound capability in enhancing smooth muscle motility via the upregulation of 5-HT receptors and the stem cell factor SCF/c-kit signaling pathways (Wang Z. et al., 2023).

As a functional subclass of non-digestible polysaccharides, adequate dietary fiber intake expands stool volume and reduces hardness, creating a positive feedback loop along the “dietary fiber–gut microbiota–intestinal motility” axis. Soluble dietary fibers, such as *hawthorn* pectin and *corn* husk fiber, undergo rapid colonic fermentation to activate G protein-coupled receptors (GPCRs), strengthening mucosal barrier integrity and promoting smooth muscle contraction (Li et al., 2025). When administered to individuals with chronic functional constipation, these specialized fiber fractions actively reshape the microbial community structure and enhance nutrient absorption, presenting a safe approach for dietary interventions.

### 4.2 Phenolic

Phenolic compounds are among the most widely distributed and structurally diverse active components of plant secondary metabolites, and their antioxidant, anti-inflammatory, and signal-regulatory functions have been confirmed by a large number of *in vitro* and *in vivo* experiments (Winiarska-Mieczan et al., 2024). Free phenols are rapidly solubilized in the gastro-small intestinal compartment and absorbed epithelially, directly reducing intracellular oxidative stress levels and thus providing primary prevention of chronic diseases (de Araújo et al., 2021). However, more than 90% of dietary polyphenols need to be transformed by the colonic microbiota: dehydroxylases, reductases, and lyases secreted by the bacteria metabolize them into more bioavailable small molecules such as urocalcitonin A and intestinal lactones, and at the same time, these metabolites can inversely shape the structure of the bacterial flora - selectively enriching commensal bacteria such as *Lactobacillus*, *Bifidobacterium*, and inhibiting potentially pathogenic bacteria (e.g., *Bacteriophages*). and *bifidobacteria*, and inhibit the colonization of potential pathogenic bacteria (Spencer and Hu, 2020). Aresearch was conducted to evaluate the intervention effect of soft date kiwifruit polyphenol (AKP) using loperamide-induced functional constipation mice as a model. The results showed that AKP significantly reversed the loperamide-induced reduction in the number of colonic Cajal interstitial cells (ICCs) and restored their intracellular Ca^2+^ homeostasis, thereby improving gastrointestinal pacing function (Sanders, 2019). At the mechanistic level, AKP promotes colonic propulsive motility by enhancing the electrical coupling between ICCs and smooth muscle through upregulation of SCF/c-Kit signaling axis activity (Liu et al., 2021). Behavioral and physiological indexes were improved simultaneously: mice in the AKP group gained faster body weight, the number of fecal pellets and water content were increased by about 138.5% and 106.5%, respectively, the whole intestinal transit rate was increased by 45.3%, and the time to discharge the first black feces was shortened by 57.5%, and the constipation symptom was significantly alleviated (Li et al., 2019).

Polyphenols exhibit a dual advantage by acting as rapid antioxidants and facilitating synergistic interactions with the gut microbiota (Bian et al., 2020). They promptly neutralize free radicals in the upper gastrointestinal tract, and following microbial metabolism, they contribute to the restoration of interstitial ICCs in both number and function via the stem cell factor SCF/c-Kit signaling pathway (Wang et al., 2017b). This process enhances gastrointestinal motility and promotes an optimal microbial community structure. Consequently, this mechanism offers a safe and viable approach for precision nutritional interventions targeting functional constipation.

### 4.3 Quinones and anthraquinones

Quinones, particularly anthraquinones, represent another critical class of bioactive secondary metabolites in specific laxative MFH plants that do not fit into the carbohydrate or phenolic categories (Wang D. et al., 2021). The most representative compound in this domain is sennoside A, a natural anthraquinone monomer derived from *Rhubarb*. Unlike prebiotics that act via slow fermentation, anthraquinone glycosides possess distinct multi-target pharmacological mechanisms. Emerging evidence demonstrates that standard doses of sennoside A significantly reduce gastrointestinal transit time and alter colonic water flux by directly modulating aquaporins aquaporin 1 (AQP1) and AQP3 (Kon et al., 2014). Simultaneously, from a microecological perspective, anthraquinones shape the bacterial community structure by selectively enriching beneficial acidifying clusters such as *Lactobacillus* while suppressing opportunistic pathogens (Takayama et al., 2021). These compounds exert rapid prokinetic effects, though their prolonged clinical application requires careful titration to avoid potential long-term colonic adaptation.

As research in this field has advanced, an increasing number of polysaccharides and polyphenols have been incorporated into clinical applications. However, the majority of medicinal food products remain at the preclinical research stage. A summary of medicinal foodstuffs with demonstrated efficacy in the prevention and management of FC is presented in Table 2.

**TABLE 2:** The effect of active ingredient from medicine food homology plants in relieving FC.

Botanical name	Active ingredient	Research target	Changes in bacterial flora	Mechanism
*Astragalus* (Liu et al., 2024)	Polysaccharide	Mice	*Acidifying bacteria*↑	Regulates the brain-gut axis
*Holothuria leucospilota* (Wang Z. et al., 2023)	Polysaccharide	Mice	*Lactobacilli*↑ *Norank-f-Muribaculaceae*↑	Promote gastrointestinal motility
*Platycodongrandiflorum* (Hao et al., 2023)	Polysaccharide	Rats	*Clostridium tumefaciens*↑	Activation of 5-HT receptors
*Chrysanthemum morifolium* (Wang J. et al., 2021)	Polysaccharide	Rats	*Lactobacilli↑, Robsonell↑*	Regulation of RAS, FABP1, and SLC1A5 protein expression
*Cranberry* (Lessard-Lord et al., 2024)	Phenol	Healthy adults	*Bifidobacterium*↑	Prebiotics and antimicrobial synergy
*Grape* (Koyama et al., 2022)	Phenol	Healthy adults	*Prevotella, Klebsiella↓, Mycobacterium, Ruminalococcus↑*	/
*Sargassum* (Khuituan et al., 2022)	Dietary fiber	Mice	*Bifidobacterium↑*	Regulates electrolyte secretion and promotes colonic contraction
*Konjac* (Hu et al., 2021)	Dietary fiber	Rats	/	Upregulation of SCF/c-kit pathway to promote intestinal motility
*Hawthorn* (Zhang et al., 2024)	Dietary fiber	Mice	*Lactobacilli ↑*	Upregulation of water channel proteins to improve inflammation
*Rhubarb* (Gao et al., 2021)	Sennoside A)	mice	*Lactobacillus↑*	Modulates AQP1/AQP3 expression and enhances fecal water content

## 5 Exploring the use of MFH in the treatment of functional constipation

MFH have garnered widespread attention in recent years as an integral part of TCM. Not only do they offer rich nutritional value and health benefits, but they are also considered to hold great potential for the development of novel pharmaceuticals. Given their high safety profile and wide range of applications, these substances are well-suited for use in functional foods, health supplements and the promotion of healthy diets, and thus hold excellent prospects for future development. Substances derived from food and medicine share common origins offer unique benefits in regulating the balance of the gut microbiota. By holistically regulating bodily functions and improving the intestinal microenvironment, they effectively treat constipation.

### 5.1 Single-ingredient MFH properties

*Rhubarb* is a commonly used remedy for constipation. It can significantly reduce the time it takes for food to pass through the digestive system and increase the water content of stools. It can also help protect the colon’s mucous layer by regulating the gut microbiota (Yang et al., 2022). Furthermore, *rhubarb* contains sennoside A, a natural anthraquinone compound. Research has found that standard doses of sennoside A, administered for less than a week, can effectively alleviate constipation symptoms in mouse models. This is primarily achieved by increasing the abundance of beneficial gut bacteria such as *Lactobacillus* and *Ijowziowtob*, whilst reducing the abundance of harmful bacteria. It also modulates the expression of the colonic aquaporins AQP1 and AQP3. However, it is worth noting that sennoide A should not be used for prolonged periods, as its laxative effect may weaken and potentially impair colonic function (Zhang et al., 2023).

*Astragalus* is a nutrient-rich plant and well-known herbal remedy, reputed to possess tonic properties. It has been widely used in China and Southeast Asia for thousands of years to treat a variety of ailments (Zhang et al., 2021). Studies have found that long-term administration of *Astragalus* can significantly modulate the gut microbiota of laying hens by increasing the abundance of *Lactobacillus*. Astragaloside IV is one of the active components of *Astragalus*. Studies have shown that in mice with loperamide-induced slow-transit constipation, Astragaloside IV effectively promotes intestinal motility by inhibiting the loss of ICCs, regulating the composition of the gut microbiota, and promoting butyrate production (He et al., 2020). *Astragalus* polysaccharides are also one of the active components of *Astragalus*. It has been reported that they can effectively prevent constipation by altering the gut microbiota and improving the intestinal environment (Liu X. et al., 2022). Microbiome analysis revealed that *Astragalus* polysaccharides increased the relative abundance of *Bacteroides* in elderly constipated rats, whilst decreasing the relative abundance of *Lactobacillus*. Furthermore, *Astragalu*s polysaccharides reduced the levels of acetic acid, butyric acid and propionic acid in faecal samples, thereby modulating glycolysis/gluconeogenesis and pyruvate metabolism.

### 5.2 Compound MFH sources

Xiao Chengqi Decoction is an ancient traditional herbal formula that has been used for thousands of years to clear heat, promote bowel movement and relieve constipation. It consists of three medicinal ingredients that are also used as food: *rhubarb*, *triturated bitter orange peel* and *magnolia bark* (Zhou et al., 2022). Found that Xiao Chengqi Decoction improves bowel movements in patients with functional constipation by protecting ICCs activity, promoting the colonisation of *Bifidobacterium*, and increasing levels of beneficial metabolites such as butylaminophenol. Furthermore, Liu et al. suggest that the anti-inflammatory activity of Xiao Chengqi Decoction is at least partly mediated by the metabolism of gut bacteria (Liu et al., 2018).

Shouhuai Tongbian Capsules are a traditional Chinese medicine widely used to treat constipation. The formulation of Shouhuai Tongbian Capsules consists of eight herbal ingredients: *Polygonum multiflorum*, *Cassia seeds*, *Lycium barbarum*, *Panax ginseng*, *Aloe vera*, *donkey-hide gelatin*, *Citrus aurantium*, and *Atractylodes macrocephala* Lin et al. (2022). Found that Shouhuai Tongbian Capsules improved the progression of loperamide-induced constipation in rats by reshaping the gut microbiota composition and regulating the production of gut metabolites. Notably, they increased the relative abundance of *Lactobacillus* and the ratio of *Firmicutes* to *Bacteroides* (F/B). Furthermore, Shouhui Tongbian Capsules alleviate constipation by regulating gut microbiota dysbiosis, modulating microbial metabolites, stimulating the release of 5-HT in the gut, and protecting the differentiation of intestinal neurons.

## 6 Shortcomings and prospects

Traditional evaluation systems fail to replicate the dynamic, multi-compartmental nature of the human digestive tract. Static *in vitro* digestion models cannot capture the temporal-spatial interactions between MFH components, mucosal barriers, and regional microbial niches. Moreover, the field slow to adopt advanced food material processing and delivery technologies, leading to poor targeted colonic delivery and low bioavailability of fragile bioactives (such as volatile polyphenols or specific oligosaccharides) during upper gastrointestinal transit.

Advancing the field of MFH-based interventions necessitates the integration of cutting-edge biomimetic and engineering technologies. The deployment of microfluidic gut-on-a-chip platforms, concurrently coupled with automated dynamic *in vitro* human digestion simulators, offers an unprecedented high-throughput paradigm for the precise screening and bioactivity profiling of MFH-derived fractions. To optimize therapeutic efficacy against FC, the development of advanced colon-targeted encapsulation delivery systems-including stimuli-responsive polysaccharide hydrogels and intelligent nano-vehicles-is imperative to safeguard labile bioactives against upper gastrointestinal degradation and gastric acidity. Ultimately, by synergetically converging microecological big data with tailored nutritional strategies, the food industry can engineer next-generation personalized functional foods meticulously tailored to the distinct enterotypes and specific microecological consortia of FC patients.

## Glossary

5-HT: 5-hydroxytryptamine
5-HT4R: 5-hydroxytryptamine receptor 4
ACh: Acetylcholine
AKP: Date kiwifruit polyphenol
ANS: Autonomic nervous system
AQP1: aquaporin 1
AQP3: aquaporin 3
AQP4: aquaporin 4
AQP8: aquaporin 8
ASBT: Apical sodium-dependent bile acid transporter
BAs: bile acids
BGA: brain-gut axis
BGBA: brain-gut-bacteria axis
**CH**_**4**_: Methane
**Cl**_**−**_: Chloride ion
CNS: Central nervous system
DA: Dopamine
ECCs: Enterochromaffin cells
ECs: Enteroendocrine cells
ENS: Enteric nervous system
FC: Functional constipation
FDD: Functional defecation disorder
FFAR2/3: Free fatty acid receptors 2 and 3
GAS: Gastrin
GPCRs: G protein-coupled receptors
**H**_**2**_ **S**: Hydrogen sulfide
HPA: Hypothalamic-pituitary-adrenal
IBS-C: Irritable bowel syndrome with constipation
ICCs: Interstitial cells of Cajal
KATP: ATP-sensitive potassium channels
MFH: Medicinal-food homology
MTL: Motilin
NO: Nitric oxide
OIC: Opioid-induced constipation
PCR: Polymerase chain reaction
PGE2: Prostaglandin E2
qPCR: Quantitative polymerase chain reaction
RCTs: Randomized controlled trials
SCFAs: Short-chain fatty acids
SP: Substance P
SS: Somatostatin
STC: Slow transit constipation
TCM: Traditional Chinese Medicine
TGR5: G protein-coupled bile acid receptor 1
TPH1: Tryptophan hydroxylase 1
TRPA1: Transient receptor potential A1
VIP: Vasoactive intestinal peptide
